# Reduced n-3 and n-6 PUFA (DHA and AA) Concentrations in Breast Milk and Erythrocytes Phospholipids during Pregnancy and Lactation in Women with Obesity

**DOI:** 10.3390/ijerph19041930

**Published:** 2022-02-09

**Authors:** Rodrigo Chamorro, Karla A. Bascuñán, Cynthia Barrera, Jorge Sandoval, Claudia Puigrredon, Rodrigo Valenzuela

**Affiliations:** 1 Department of Nutrition, Faculty of Medicine, University of Chile, Santiago 8320000, Chile; rchamorro@uchile.cl (R.C.); kbascunan@uchile.cl (K.A.B.); cbarrera@uchile.cl (C.B.); 2 Obstetrics and Gynecology Department, Clinical Hospital of the University of Chile, Santiago 8320000, Chile; josandoval@med.uchile.cl (J.S.); cpuigrredon@hcuch.cl (C.P.)

**Keywords:** obesity, pregnancy, lactation, maternal diet, breast milk, arachidonic acid, docosahexaenoic acid

## Abstract

Obesity during pregnancy is a worrying public health problem worldwide. Maternal diet is critical for fatty acid (FA) placental transport and FA content in breast milk (BM). We evaluated FA composition in erythrocytes phospholipids (EP) and BM in pregnant women with (OBE, n = 30) and without (non-OBE, n = 31) obesity. Sixty-one healthy women were evaluated at their 20–24th gestational week and followed until 6th month of lactation. Diet was evaluated through a food frequency questionnaire. FA composition of EP and BM was assessed by gas-liquid chromatography. The OBE group showed lower diet quality, but total n-6 and n-3 polyunsaturated FA (PUFA), ALA, EPA, and DHA dietary intake was similar between groups. N-3 PUFA, ALA, DHA, and the n-6/n-3 PUFA ratio in EP were lower at the 6th lactation month in the OBE group. In BM, the arachidonic acid (AA) concentration was lower at the end of the lactation, and DHA content showed an earlier and constant decline in the OBE group compared to the non-OBE group. In conclusion, n-3 PUFA and AA and DHA levels were reduced in EP and BM in pregnant women with obesity. Strategies to increase n-3 PUFA are urgently needed during pregnancy and lactation, particularly in women with obesity.

## 1. Introduction

Maternal nutrition during pregnancy and lactation is relevant for fetal and infant human development [1]. An adequate nutrient supply from mother-to-fetus and mother-to-newborn is critical for short- and long-term health outcomes of offspring [2,3]. Maternal diet and nutrition before conception, during pregnancy and breastfeeding, are essential to ensure adequate nutrients supply to the fetus and infant [4]. The association of perinatal maternal morbidity with preconception obesity has been well established. The effects of maternal obesity in utero range from fetal growth, neonatal body composition, and later adolescent obesity [5,6].

Obesity during pregnancy is a rising public health problem in different societies [6] and is rapidly increasing worldwide [7,8,9,10]. Excessive weight gain in this period is common (about 50% of pregnant women) [9] and is consistently associated with adverse fetal and child’s metabolic and neurocognitive outcomes [11,12]. Moreover, a higher risk for macrosomia, newborns large for gestational age, cesarean delivery, and maternal hypertension alterations have been reported [13,14]. Obesity is common during pregnancy, affecting both the mother and her offspring [15]. It causes short- and long-term problems for the mother, such as the increased risk of excessive gestational weight gain (GWG), gestational diabetes (GDM), pre-eclampsia, and cardiovascular diseases [16]. In the offspring, maternal obesity increases prematurity risk, fetal death, injury during birth, transient respiratory problems, and metabolic effects (i.e., insulin resistance in utero [17] and neonatal hypoglycemia) [18]. Maternal obesity also predisposes the offspring to long-term health problems, potentially generating an intergenerational cycle of obesity and insulin resistance [19,20].

Diet plays a critical role during pregnancy, and a high energy intake through high-sugar and high-saturated fat foods (energy-dense foods) contribute to excessive weight gain through this period [4]. During breastfeeding, maternal diet and body stores accumulated during pregnancy will influence—among others—the fatty acids (FA) composition of breast milk, particularly the content of n-6 and n-3 polyunsaturated fatty acids (PUFA) [21]. These FA have a critical role in human biochemistry and physiology [22]. In breast milk, arachidonic (C20:4n-6, AA) and docosahexaenoic (C22:6n-3, DHA) acids are two of the most important PUFA for infant development [23,24]. Specifically, DHA and AA give flexibility and fluidity to the neuronal plasma membrane, facilitating the signal transduction process, neuronal growth and migration, synaptogenesis, and synaptic plasticity [25]. In addition, these PUFA can modulate energetic metabolism in muscle and adipose tissue, such as insulin action and inflammatory response [26].

The supply of AA and DHA to the fetus and the newborn can originate from: (i) mother’s FA stores, (ii) endogenous maternal synthesis or (iii) directly from the maternal diet [27]. Obesity during pregnancy and lactation could lead to (i) decreased hepatic PUFA synthesis [28] or (ii) reduced circulating PUFA levels [29]. It has been demonstrated that placental uptake of PUFA (linoleic acid and DHA) was lower in women with obesity, suggesting a reduced capacity for materno-fetal long-chain PUFA transfers in this group of women [30]. We have reported that the maternal diet of healthy women during pregnancy and breastfeeding characterizes by a deficient intake of n-3 PUFA (especially DHA) [31]. This dietary insufficiency was reflected in decreased DHA content of breast milk at 6th postpartum month [31]. Here, we aimed to evaluate FA composition in erythrocytes (during pregnancy and lactation) and breast milk (from first to sixth months of lactation) in pregnant women with (OBE) and without obesity (non-OBE).

## 2. Material and Methods

### 2.1. Study Design and Participants

This is an observational study including sixty-one pregnant women attending at the Obstetrical and Gynecology Unit, Clinical Hospital of the University of Chile. We included adult pregnant women with an age range between 20 and 35 years, gestational age between 22 and 24 weeks (according to the date of the last menstrual period and confirmed by ultrasound), with a previously successful and healthy pregnancy (having a single term and no more than two newborns) and having successful lactation in previous pregnancies. As exclusion criteria, we considered women with a history of drugs or alcohol consumption, a regular intake of PUFA supplements, being underweight (defined by the Chilean charts for pregnant women [32]), having experienced a previous twin pregnancy, chronic diseases, such as type 2 diabetes, hypertension, and any illness or medical condition that could affect normal fetal growth.

The study protocol was reviewed and approved by the Institutional Review Board of the Faculty of Medicine, University of Chile (Protocol #073-2011) and by the Ethics Committee of the Clinical Hospital, University of Chile (Protocol #507/11). All information about the study was given to each participant who voluntarily agreed to participate. Women read and signed the written informed consent.

### 2.2. Clinical and Nutritional Evaluation

When incorporated into the study, women underwent a clinical evaluation when incorporated into the study (~22 gestational weeks). A physician and a trained nurse evaluated each woman as part of the regular health controls following the national clinical protocol. Bodyweight (kg) and height (m) were evaluated twice with women using light clothes, and body-mass index (BMI, kg/m^2^) was then calculated. Maternal nutritional status was evaluated using BMI according to gestational weeks [32]. The use of the body-mass index (BMI) was validated at the national level to assess pregnant women’s nutritional status. In brief, Chilean charts for pregnant women adjust BMI classification according to gestational weeks. The chart validated the use of BMI cut-off points in the context of gestational weight gain data associated with a lower risk of maternal and fetal morbidity [32].

Two groups of pregnant women were studied: a group with obesity (OBE, n = 30) and a group of normal-weight women (non-OBE, n = 31) according to gestational weeks [32]. Energy and nutrient requirements were calculated following WHO criteria [33] and recommended daily dietary intake followed the American Institute of Medicine criteria (2001) [34]. All women underwent a complete nutritional assessment, including nutritional diagnosis and counseling for a healthy diet following national dietary guidelines [35].

### 2.3. Dietary Intake

Dietary intake was evaluated through a food-frequency questionnaire (FFQ) applied by two trained nutritionists. FFQ was applied at the first clinical evaluation (between 20 and 24th gestational week), during the first week after delivery, and at the 6th lactation month. Each woman was interviewed and asked to report all foods consumed during the last month. Nutritionists used the “Atlas of Commonly Consumed Foods in Chile”, a photographic tool for a precise estimation of serving portions of each food/beverage [36]. Dietary data were checked by contrasting energy/nutrient intake data with dietary questionnaires, identifying missing information and potential outliers. A careful review of each FFQ was done, and, if needed, participants were contacted, and data was checked.

According to the dietary analysis previously described by our workgroup [37], data were grouped into nine food groups (cereals, fruits and vegetables, dairy, meats and eggs, legumes, fish and shellfish, high-lipid foods, oils and fats, and sugars and processed foods). Daily consumption of each reported food/beverage was calculated, and dietary composition was analyzed using Food Processor SQL^®^ (ESHA Research, Salem, OR, USA). We also used a locally generated database, including nutrient composition data from manufacturer labeling of foods and preparations commonly used in Chile. Energy and nutrient intake were estimated using a food database from the USDA National Nutrient Database for Standard Reference.

### 2.4. Blood and Breast Milk Samples

Blood samples were obtained at the 6th month of pregnancy, and the 1st and 6th month of lactation. Butylated hydroxytoluene (BHT) was added to blood samples as an antioxidant, and samples were immediately centrifuged. The erythrocyte fraction was obtained (3000× *g* for 10 min at 20 °C). After, samples were frozen at −80 °C for further analysis. During the first six months of lactation, breast milk (5 mL) samples were obtained monthly and collected in plastic vials after newborns had been fed for at least 2 min; samples were immediately frozen at −80 °C for further analysis.

### 2.5. Lipids Extraction from Erythrocytes and Breast Milk

Quantitative extraction of total lipids from erythrocytes phospholipids and breast milk was carried out according to Bligh and Dyer [38], with the addition of Butylated hydroxytoluene (BHT). Erythrocytes and breast milk samples were mixed separately with ice-cold chloroform/methanol (2:1 *v*/*v*, containing 0.01% BHT). Magnesium chloride was added (0.5 N) and the mixture homogenized in an Ultraturrax homogenizer (Janke & Kunkel, Stufen, Germany). Total lipids from erythrocytes and milk samples were separated by thin-layer chromatography (TLC), as reported previously [39].

### 2.6. Gas Chromatography Analysis of Fatty Acid Methyl Esters

Fatty acid methyl esters (FAMEs) from erythrocyte phospholipids and milk FA were prepared according to Morrison and Smith [40]. Samples previously dissolved in chloroform/methanol (2:1 *v*/*v*) were then evaporated under nitrogen stream until the volume was halved. Boron trifluoride (12% methanolic solution) and sodium hydroxide (0.5 N methanolic solution) were added, after which the mixture was cooled. FAMEs were extracted with 0.5 mL of hexane. Details of the gas chromatographic analysis of FA from erythrocyte phospholipids and breast milk samples were previously described by Valenzuela et al. [41]. FA level was expressed as moles %.

### 2.7. Statistical Analysis

After descriptive analysis, the distribution of variables was evaluated using the Shapiro–Wilk test and visual inspection. Assessment of significant differences between groups regarding diet and FA composition were evaluated using Student’s *t*-test for unpaired data or one-way analysis of variance (ANOVA), with Bonferroni as post hoc test (*p* < 0.05). The association between DHA and AA concentration in breast milk samples and BMI was evaluated through Pearson’s correlation test. Data are expressed as mean ± SD. Statistical significance was set at a 5% alpha level. The software SPSS v.24.0 (Chicago, IL, USA) was used in all analyses.

## 3. Results

### 3.1. Background Characteristics

Table 1 shows the main background characteristics of participants. Women were mainly young (age range between 20 and 35 years). As expected by the study design, preconception weight and BMI were higher in the obese group. GWG, estimated as the difference between body weight at delivery and preconception weight (in kg), was not different between groups. Anthropometrical differences remain significant between groups at the 6th pregnancy month and at the 1st and 6th lactation months. Gestational weeks showed no differences between groups during pregnancy and at delivery.

### 3.2. Food Groups Consumption

Comparison of food groups consumption between OBE and non-OBE groups showed several differences. Lower fruits/vegetables and dairy intake were observed in the OBE group during all evaluated periods (Table 2). The same group also showed higher consumption of high-fat foods, oils and fats, and sugar and processed foods. No between-groups differences were observed regarding other food groups (Table 2).

### 3.3. Energy, Nutrients, and FA Dietary Intake

Table 3 shows energy, nutrients, and the most relevant fatty acids dietary intake. The OBE group showed consistently higher energy intake across all three periods than the non-OBE group. Total protein and carbohydrate intake were similar, but the OBE group’s sugar intake was higher. In all evaluated periods, total fat intake was significantly higher in the OBE group, mainly due to a higher saturated fatty acids (SFA) intake (Table 3). Total n-6 and n-3 PUFA and alpha-linolenic acid (C18:3n-3, ALA), eicosapentaenoic acid (C20:5n-3, EPA), and DHA intake were similar between groups.

### 3.4. Fatty Acid Composition of Erythrocyte Phospholipids

Table 4 shows the FA composition of erythrocyte phospholipids. No significant differences were found for SFA, monounsaturated fatty acids (MUFA), PUFA, n-6 PUFA, linoleic acid (C18:2n-6, LA), and n-6 docosapentaenoic acid (C22:5n-6, DPAn-6) n-6 when comparing FA concentration between the 1st lactation month and the 6th lactation between OBE and non-OBE groups. However, AA concentration was reduced in the OBE group at the 6th lactation month. N-3 PUFA, ALA, DHA, and the n-6/n-3 PUFA ratio was significantly lower in the OBE group for all studied periods. In addition, EPA and docosapentaenoic acid (C22:5n-3, DPAn-3) showed reduced levels in the OBE group, particularly at the 6th lactation month (Table 4).

### 3.5. Fatty Acid Composition of Breast Milk

**Figure 1:**
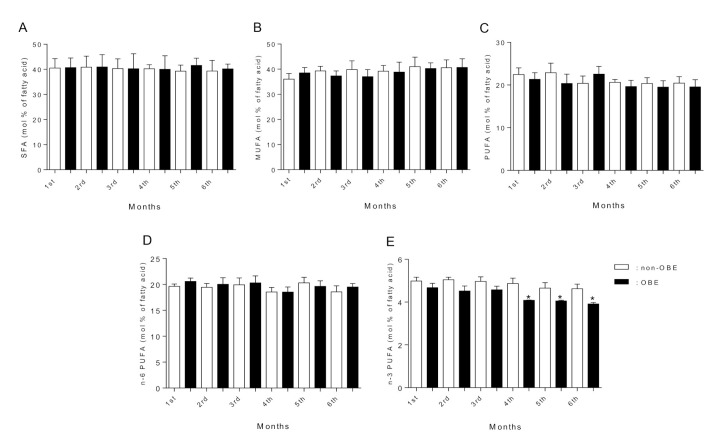
(**A**) SFA; (**B**) MUFA; (**C**) PUFA; (**D**) n-6 PUFA, and (**E**) n-3 PUFA content in breast milk. Data as mean ± SD and expressed as mol % of fatty acid (non-OBE, n = 31, and OBE, n = 30); one-way ANOVA with Bonferroni post hoc test. Columns without a symbol or that share the same character are not significantly different from each other. * Indicates statistically significant differences with the other groups. Non-OBE: women without obesity; OBE: women with obesity.

**Figure 2:**
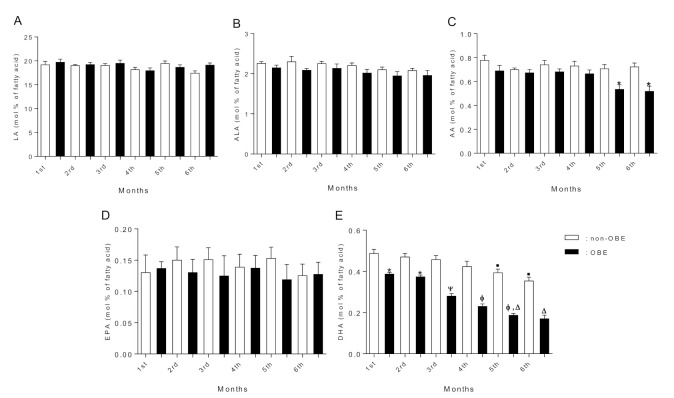
(**A**) LA; (**B**) ALA; (**C**) AA; (**D**) EPA, and (**E**) DHA content in breast milk. Data as mean ± SD and expressed as mol % of fatty acid (non-OBE, n = 31, and OBE, n = 30); one-way ANOVA with Bonferroni post hoc test. Columns without a symbol or that share the same character are not significantly different from each other. *, Ψ, Φ, ▪ or Δ symbols Indicates statistically significant differences with the other groups. Non-OBE: women without obesity; OBE: women with obesity.

**Figure 3:**
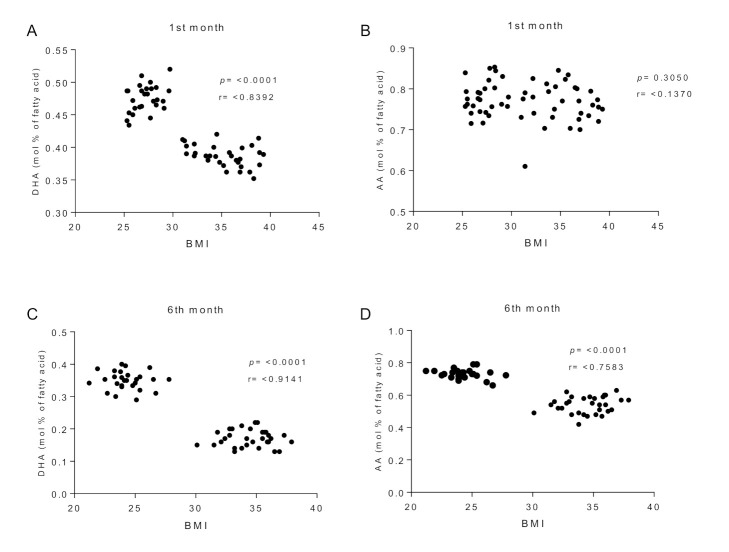
Correlations between DHA and AA breast milk content at 1st and 6th months with body-mass index (BMI). (**A**) DHA content v/s BMI at 1st lactation month; (**B**) AA content v/s BMI at 1st lactation month; (**C**) DHA content v/s BMI at 6th lactation month; (**D**) AA content v/s BMI at 6th lactation month.

Figure 1 and Figure 2 show the FA composition in breast milk. We found rather similar SFA, MUFA, and total PUFA levels across lactation between both groups (Figure 1A–D). However, n-3 PUFA content at the 4th and 6th lactation months was significantly lower in the OBE group (Figure 1E). N-6 and n-3 fatty acids (LA, ALA, and EPA, Figure 2A,B,D, respectively) concentrations showed similar levels between groups. AA concentration was lower at the OBE group’s 5th and 6th lactation months (Figure 2C). Notably, DHA concentration in breast milk was significantly lower across the whole lactation period in the OBE group (Figure 2E). DHA content in breast milk showed significant differences across lactation in the OBE group, with a constant and significant DHA decrease (Figure 2E). Starting at the 3rd month, DHA concentration was significantly lower compared with the 1st and 2nd months (*p* < 0.05). Differences remained until the 6th lactation month, when DHA was still lower than the 1st and 4th months (Figure 2E). The same analysis in the non-OBE group showed reduced DHA content only at months 5 and 6 compared with the previous months (Figure 2E). Finally, correlation analysis showed an inverse association (r = −0.83, *p* < 0.0001) between DHA (but not AA) content in breast milk and BMI at the 1st lactation month (Figure 3A,B). The same association was found for both DHA (r = −0.91, *p* < 0.0001) and AA (r = −0.75, *p* < 0.0001) content in breast milk and BMI at the 6th lactation month (Figure 3C,D).

## 4. Discussion

We aimed to evaluate FA composition in erythrocytes and breast milk in a group of pregnant women with and without obesity from the last trimester of pregnancy until the 6th lactation month. Our main findings show that the content of n-3 PUFA and DHA in erythrocyte phospholipids was significantly lower during both pregnancy and lactation in women with obesity. The content of AA and EPA in erythrocyte phospholipids was also reduced at the 6th lactation month in this group. In addition, n-3 PUFA and AA content in breast milk was reduced at the 5th and 6th lactation months in the OBE group. DHA content was consistently lower across the whole lactation period in women with obesity compared with their normal-weight peers.

The worldwide prevalence of obesity has been rising during the last decades. A significant increase in overweight and obesity among pregnant women has been reported in both high-income and middle-income countries [8,42]. In this context, studies suggest that Chilean childbearing women show a fast and growing tendency towards obesity [43], and a worryingly high prevalence of pregnant women with overweight and obesity was observed [44]. Moreover, maternal obesity is related to several health consequences for the mother and infant [12,13], increasing the infant’s risk of developing several chronic diseases such as obesity, type 2 diabetes, and cardiovascular diseases [45], and increasing body fat in male 2–6 years children [46]. Our results show that women with obesity displayed an impaired FA profile in erythrocytes and breast milk—compared to a group of normal-weight women—during the last part of pregnancy and the first six months of lactation.

Concerning FA metabolism, obesity during pregnancy poses several adverse consequences, as well. Studies have shown an imbalance in dietary intake of n-6 and n-3 PUFA in women with obesity mainly increased n-6 PUFA and decreased n-3 PUFA [31,47]. On the other hand, maternal obesity during pregnancy and lactation can alter PUFA metabolism, leading to decreased PUFA levels and synthesis [28,29] and impaired capacity for materno-fetal long-chain PUFA transfer [30]. It has also been shown that placental transfer of FA transporters is impaired by obesity [48], leading to reduced PUFA supply to the fetus [30]. The endogenous synthesis of DHA from ALA can be affected by obesity itself and obesity-related metabolic diseases [49], reducing the activity of Δ-5 and Δ-6 desaturase enzymes [28], leading to impaired DHA synthesis and lower content of n-3 long-chain PUFA in erythrocytes and breast milk. In this regard, the PUFA synthesis process is directly related to Δ-6 and Δ-5 desaturase enzymes activity (particularly in the liver) [50]. This enzymatic pathway process can be modulated by liver steatosis induced by a high-fat diet (with high SFA content, such as palmitic acid) or oxidative stress [49,50], common metabolic conditions found in obesity [49]. In line with our results, the lower n-3 PUFA, DHA, and AA levels in erythrocytes and breast milk in the OBE group, could be explained by the alteration in the FA desaturation capacity. This is supported by the similar dietary intake of AA, ALA, DHA, and EPA between non-OBE and OBE groups and by the fact that the OBE group showed higher SFA intake than their normal-weight peers (Table 3).

Breast milk provides several nutrients, immune cells, and bioactive molecules during the lactation period for optimal infant growth [51,52]. An adequate supply of DHA and AA through breastfeeding is of paramount relevance for an infant’s nutrition and development [53]. DHA and AA content in human milk depends on maternal diet [54,55,56]. In this regard, humans can synthesize PUFA from LA and ALA, both being essential precursors of more unsaturated and long-chain FA [57]. LA is the precursor of AA [58], whereas ALA is the precursor of EPA and DHA, two of the most representative FA of the n-3 PUFA family [58]. The synthesis of PUFA is an important process that occurs mainly in the liver and provides a constant flow of these FA to cells and tissues, such as the brain, retina, and immune system cells [59]. PUFA synthesis is dependent on the availability of substrate (LA or ALA), activity of the elongases and desaturases enzymes (e.g., Δ-5 and Δ-6 desaturases), availability of specific nutrients and the redox state (particularly in the liver) [59,60,61].

The relevance of preventing obesity during pregnancy is highlighted by several lines of evidence showing a significant impact on the fetus and infant development. Studies implementing PUFA supplementation have shown positive results improving FA maternal status [25] but more inconsistent results concerning metabolic regulation and infant body composition [62,63]. In this regard, the low intake of n-3 long-chain PUFA, as seen in our study, is critical for nutritional intervention. Dietary strategies have been implemented to improve women’s diet quality during pregnancy and lactation through educational programs focused on promoting the consumption of foods that provide DHA [64,65]. It is known that it is not easy to modify dietary habits, but pregnancy is when dietary habit modifications can achieve improved results [9]. Several studies have used nutritional supplements containing DHA or DHA-added foods, evidencing that an increase in the intake of DHA increases the content of this FA in erythrocytes and subsequently in breast milk in pregnant and lactating women [66,67]. Therefore, dietary strategies focused on increasing weekly fish consumption can improve maternal PUFA status with potential benefits for the fetus and the infant. However, when obesity is present, the results from these interventions can be attenuated, as women with obesity show a lower increase in n-3 FA levels and a lower reduction in the n-6:n-3 ratio after supplementation compared with normal-weight women [68].

As limitations, we assessed dietary intake using dietary surveys (FFQ), a method that possesses limitations [69]. However, we have used this instrument previously, and it has been validated in this population with the focus on dietary sources of fat and fatty acid intake [31,37,41]. We used a second instrument (24-h recall) to corroborate dietary structure and the dietary pattern of studied women, complementing the data from the FFQ. The fact that food information from dietary surveys is based on food composition data is another plausible limitation [70]. Several factors have been reported to potentially affect food composition data, including food sampling processes, as well as sources of analytical, environmental, and biological variability [70,71]. We used the USDA National Nutrient Database for reference and included manufacturer and local food composition data for specific national products. This last point allowed us to complement nutritional information with local foods commonly consumed by the Chilean population. Regarding strengths, we can mention that we carried out a complete analysis of the composition of FA in erythrocytes and breast milk, both components that provide complementary information regarding the FA composition status. In addition, the erythrocyte and breast milk measurements and the dietary analysis based on the FFQ allowed us to comprehensively determine the FA status.

## 5. Conclusions

Maternal obesity is related to the Western diet, characterized by the higher content of energy, SFA, and sugars. These dietary changes increase adipose tissue mass, metabolic disturbances (such as insulin resistance, inflammation, and oxidative stress), and the alteration of PUFA synthesis. Our results show that dietary n-3 PUFA, DHA, and AA concentration in erythrocytes phospholipids and breast milk are reduced in women with obesity during the last part of pregnancy and the first six months of lactation. Dietary strategies are urgently needed to improve diet quality in pregnant and lactating women with obesity. These strategies should focus on incrementing n-3 PUFA status (especially DHA) from foods and supplements.

## Figures and Tables

**Table 1:** Background characteristics.

	Non-OBE (n = 31)	OBE (n = 30)	*p*-Value
Age (years)	28.5 ± 4.5	27.1 ± 5.2	0.784
Preconception weight (kg)	54.7 ± 4.7	76.3 ± 6.6	0.031 *
Height (m)	1.56 ± 0.15	1.62 ± 0.17	0.684
Preconception BMI (kg/m^2^)	22.6 ± 2.2	29.1 ± 2.9	0.039 *
*At enrollment*			
Weight (kg)	62.1± 5.5	84.9 ± 7.4	0.042 *
BMI (kg/m^2^)	25.5 ± 2.4	32.5 ± 2.8	0.031 *
Gestational age (weeks)	24.5 ± 1.9	24.9 ± 2.2	0.814
*After delivery*			
Weight (kg)	66.8 ± 6.9	89.8 ± 7.2	0.035 *
BMI (kg/m^2^)	27.8 ± 1.9	34.2 ± 2.8	0.043 *
Gestational age (weeks)	39.1 ± 2.2	37.9 ± 2.4	0.793
Body weight gain (kg) ^†^	12.2 ± 4.7	13.6 ± 5.9	0.629
At 6th postpartum month			
Weight (kg)	57.6 ± 4.9	83.7 ± 6.8	0.029 *
BMI (kg/m^2^)	23.9 ± 1.8	32.3 ± 3.0	0.037 *

Data are shown as mean ± SD, or as a percentage (%); SES, socioeconomic status; BMI, body mass index = kg/m^2^; non-OBE: women without obesity; OBE: women with obesity. All data showed normal distribution. Groups were compared with Student’s *t*-test for independent samples (*p* < 0.05). * Indicates statistically significant differences. ^†^ Body weight gain (weight at delivery-preconception weight).

**Table 2:** Daily food groups intake (g/day) across pregnancy and lactation time points.

	6th Pregnancy Month		1st Lactation Month		6th Lactation Month	
Food Groups	Non-OBE ^a^	OBE ^b^	Non-OBE ^c^	OBE ^d^	Non-OBE ^e^	OBE ^f^
Cereals	298.2 ± 32.4	382.3 ± 51.0	284.7 ± 35.8	372.5 ± 45.2	271.7 ± 39.5	366.3 ± 50.4
Fruits and Vegetables	501.5 ± 47.6 ^b,d,f^	375.2 ± 32.9 ^a,c,e^	472.6 ± 58.9 ^b,d,f^	302.9 ± 31.4 ^a,c,e^	457.8 ± 38.9 ^b,d,f^	314.5 ± 25.9 ^a,c,e^
Dairy	599.7 ± 40.9 ^b,d,f^	325.6 ± 31.9 ^a,c,e^	492.7 ± 42.6 ^b,d,f^	294.5 ± 29.9 ^a,c,e^	479.2 ± 45.2 ^b,d,f^	239.4 ± 50.6 ^a,c,e^
Meats and Eggs	101.1 ± 12.5	119.4 ± 29.9	89.5 ± 19.6	107.6 ± 19.4	85.9 ± 10.8	102.8 ± 26.3
Fish and Seafood	25.3 ± 6.7	22.1 ± 6.0	28.9 ± 7.4	24.5 ± 10.5	23.5 ± 5.5	20.4 ± 6.9
Legumes	30.1 ± 8.5	18.2 ± 9.6	25.9 ± 7.1	15.4 ± 7.5	35.8 ± 10.2	20.1 ± 5.8
High-Fat Foods	25.2 ± 5.8 ^b,d,f^	56.9 ± 10.6 ^a,c,e^	23.6 ± 6.3 ^b,d,f^	50.1 ± 13.6 ^a,c,e^	20.9 ± 5.5 ^b,d,f^	52.8 ± 9.8 ^a,c,e^
Oils and Fats	28.9 ± 6.3 ^b,d,f^	44.6 ± 9.6 ^a,c,e^	25.9 ± 5.4 ^b,d,f^	40.8 ± 10.6 ^a,c,e^	23.7 ± 8.2 ^b,d,f^	41.8 ± 12.6 ^a,c,e^
Sugar and Processed Foods	161.4 ± 30.5 ^b,d,f^	402.6 ± 69.7 ^a,c,e^	182.6 ± 44.6 ^b,d,f^	384.5 ± 60.1 ^a,c,e^	170.5 ± 38.6 ^b,d,f^	371.5 ± 78.3 ^a,c,e^

Data are mean ± SD. ^a^ Significantly different to normal weight 6th month of pregnancy; ^b^ Significantly different to obese 6th month of pregnancy; ^c^ Significantly different to normal weight 1st month of lactation; ^d^ Significantly different to obese 1st month of lactation; ^e^ Significantly different to normal weight 6th month of lactation; ^f^ Significantly different to obese 6th month of lactation. Food intake was organized in nine groups according to the methodology described in the text (see methods). One-way ANOVA and Bonferroni test (*p* < 0.05). non-OBE: women without obesity; OBE: women with obesity.

**Table 3:** Energy, nutrients, and most relevant fatty acid intake during pregnancy and lactation periods.

	6th Month of Pregnancy		1st Month of Lactation		6th Month of Lactation	
Energy/Nutrients/Fatty Acid	Non-OBE ^a^	OBE ^b^	Non-OBE ^c^	OBE ^d^	Non-OBE ^e^	OBE ^f^
Energy (kcal/day)	2142 ± 274 ^b^	2895 ± 316 ^a,c,e^	1852 ± 205 ^b,d,f^	2659 ± 302 ^c,e^	1793 ± 199 ^a,b,d,f^	2438 ± 294 ^c,e^
Protein (g/day)	81.1 ± 26.5	98.5 ± 31.5	73.1 ± 24.3	90.6 ± 29.4	66.5 ± 19.2	84.5 ± 22.3
Carbohydrate (g/day)	325.3 ± 62.4	449.5 ± 72.3	247.2 ± 51.4	401.6 ± 82.9	232.1 ± 44.6	362.4 ± 80.1
Fiber (g/day)	42.7 ± 14.9 ^f^	20.1 ± 10.3	39.6 ± 12.8	18.5 ± 9.3	31.7 ± 9.2	17.2 ± 6.8 ^a^
Sugar (g/day)	90.5 ± 20.5 ^b,d,f^	198.5 ± 45.9 ^a,c,e^	82.6 ± 22.9 ^b,d,f^	184.3 ± 38.7 ^a,c,e^	72.4 ± 18.0 ^b,d,f^	175.2 ± 32.6 ^a,c,e^
Fat (g/day)	74.8 ± 11.4 ^b,d,f^	124.1 ± 21.3 ^a,c,e^	69.2 ± 14.1 ^b,d,f^	121.3 ± 19.1 ^a,c,e^	69.6 ± 15.8 ^b,d,f^	115.5 ± 22.0 ^a,c,e^
ΣSFA (g/day)	20.1 ± 2.5 ^b,d,f^	44.3 ± 3.8 ^a,c,e^	18.7 ± 2.2 ^b,d,f^	36.9 ± 3.6 ^a,c,e^	16.5 ± 2.2 ^b,d,f^	32.5 ± 3.3 ^a,c,e^
ΣMUFA (g/day)	27.6 ± 3.6	35.7 ± 3.0	25.9 ± 2.7	38.9 ± 2.9	25.1 ± 2.4	37.7 ± 2.5
ΣPUFA (g/day)	26.9 ± 2.9	42.1 ± 4.1 ^a,c,e^	24.6 ± 2.5	45.4 ± 6.7 ^a,c,e^	27.9 ± 2.8	45.1 ± 6.4 ^a,c,e^
Σ*n*-6 PUFA (g/day)	24.8 ± 2.3	38.0 ± 3.5 ^a,c,e^	21.2 ± 2.1	41.9 ± 5.1 ^a,c,e^	25.9 ± 2.5	42.2 ± 4.2 ^a,c,e^
18:2*n*-6 (LA) (g/day)	23.2 ± 2.1	36.4 ± 3.4 ^a,c,e^	21.3 ± 2.3	39.9 ± 3.7 ^a,c,e^	24.7 ± 2.2	40.8 ± 2.0 ^a,c,e^
20:4*n*-6 (AA) (g/day)	0.72 ± 0.2	0.86 ± 0.3	0.81 ± 0.2	0.71 ± 0.1	0.82 ± 0.2	0.77 ± 0.1
Σ*n*-3 PUFA (g/day)	2.05 ± 0.6	2.82 ± 0.7	1.95 ± 0.5	2.92 ± 0.8	1.92 ± 0.5	2.75 ± 0.4
18:3*n*-3 (ALA) (g/day)	1.82 ± 0.6	2.65 ± 0.7	1.71 ± 0.4	2.75 ± 0.7	1.71 ± 0.6	2.59 ± 0.4
20:5*n*-3 (EPA) (g/day)	0.05 ± 0.02	0.04 ± 0.01	0.06 ± 0.02	0.04 ± 0.01	0.05 ± 0.02	0.04 ± 0.02
22:6*n*-3 (DHA) (g/day)	0.12± 0.03	0.09 ± 0.2	0.13 ± 0.04	0.10 ± 0.02	0.11± 0.03	0.08 ± 0.02
*n*-6/*n*-3 PUFA ratio	12.1 ± 1.2	13.4 ± 1.4	11.5 ± 1.1	14.1 ± 1.8	13.4 ± 1.4	15.3 ± 1.9

Data shown as the mean ± SD; ^a^ Significantly different to normal weight 6th month of pregnancy; ^b^ Significantly different to obese 6th month of pregnancy; ^c^ Significantly different to normal weight 1st month of lactation; ^d^ Significantly different to obese 1st month of lactation; ^e^ Significantly different to normal weight 6th month of lactation; ^f^ Significantly different to obese 6th month of lactation. One-way ANOVA and Bonferroni test. (*p* < 0.05). Saturated fatty acids (SFA) correspond to C6:0, C8:0, C10:0, C12:0, C14:0, C16:0, C18:0, C20:0, C22:0, and C24:0. Monounsaturated fatty acids (MUFA) correspond to C14:1n-5, C16:1n-7, and C18:1n-9. Polyunsaturated fatty acids (PUFA) correspond to C18:2n-6, C18:3n-3, C20:4n-6, C20:5n-3, C22:5n-6, C22:5n-3, and 22:6n-3. n-6 PUFA correspond to C18:2n-6, C20:4n-6, and C22:5n-6. n-3 PUFA correspond to C18:3n-3, C20:5n-3, C22:5n-3, and C22:6n-3. non-OBE: women without obesity; OBE: women with obesity.

**Table 4:** Fatty acid composition in erythrocyte phospholipids of the women during the pregnancy and lactation period.

	6th Month of Pregnancy		1st Month of Lactation		6th Month of Lactation	
FA (Expressed as mol % of Fatty Acid)	Non-OBE ^a^	OBE ^b^	Non-OBE ^c^	OBE ^d^	Non-OBE ^e^	OBE ^f^
ΣSFA	49.8 ± 4.7	53.7 ± 5.1	48.8 ± 5.0	52.9 ± 5.6	49.3 ± 5.2	53.0 ± 5.4
C16:0	30.3 ± 3.2	33.2 ± 3.7	30.9 ± 2.9	32.9 ± 3.5	31.5 ± 3.3	34.5 ± 3.6
C18:0	15.8 ± 1.8	18.8 ± 1.9	16.1 ± 1.4	17.9 ± 1.9	16.5 ± 1.4	18.2 ± 1.9
ΣMUFA	16.8 ± 1.7	15.2 ± 1.4	17.3 ± 1.6	15.9 ± 1.7	18.3 ± 1.8	16.9 ± 1.5
C18:1*n*-9	14.5 ± 1.4	13.8 ± 1.3	15.5 ± 1.7	13.6 ± 1.4	17.3 ± 1.8	15.5 ± 1.2
ΣPUFA	33.4 ± 3.8	31.3 ± 3.2	33.9 ± 3.6	31.2 ± 3.0	32.4 ± 3.7	30.1 ± 3.3
Σ*n*-6 PUFA	24.8 ± 2.5	25.9 ± 2.9	25.8 ± 2.4	26.3 ± 2.7	24.5 ± 2.6	25.7 ± 2.8
C18:2*n*-6 (LA)	11.3 ± 1.3	12.6 ± 1.5	12.8 ± 1.9	13.5 ± 2.2	12.7 ± 1.4	13.9 ± 1.7
C20:4 *n*-6 (AA)	12.5 ± 1.6	11.9 ± 1.4	11.6 ± 1.3	11.2 ± 1.5	10.7 ± 1.2	9.61 ± 0.5 ^a^
C22:5*n*-6 (DPA*n*-6)	0.81 ± 0.1	0.79 ± 0.2	0.87 ± 0.05	0.81 ± 0.1	0.95 ± 0.2	0.92 ± 0.3
Σ *n*-3 PUFA	8.60 ± 1.0 ^b,d,f^	5.40 ± 0.4 ^a,c,e^	8.10 ± 0.9 ^b,d,f^	4.90 ± 0.5 ^a,c,e^	7.90 ± 1.1 ^b,d,f^	4.38 ± 0.4 ^a,c,e^
C18:3*n*-3 (ALA)	1.88 ± 0.3 ^b,d,f^	1.15 ± 0.2 ^a,c,e^	2.09 ± 0.5 ^b,d,f^	1.05 ± 0.3 ^a,c,e^	2.15 ± 0.6 ^b,d,f^	1.01 ± 0.4 ^a,c,e^
C20:5*n*-3 (EPA)	1.57 ± 0.4 ^b,d,f^	0.93 ± 0.1 ^a,d,f^	1.24 ± 0.3 ^f^	0.51 ± 0.2 ^a,b^	1.19 ± 0.2 ^d,f^	0.44 ± 0.1 ^a,b,c,e^
C22:5*n*-3 (DPA*n*-3)	0.58 ± 0.1 ^c,d,e,f^	0.40 ± 0.1 ^d,e,f^	0.38 ± 0.05 ^a,d,f^	0.21 ± 0.03 ^a,b,c^	0.29 ± 0.04 ^a,b,f^	0.17 ± 0.03 ^a,b,c,e^
C22:6*n*-3 (DHA)	4.53 ± 0.5 ^b,d,f^	3.04 ± 0.4 ^a,c,e^	4.39 ± 0.6 ^b,d,f^	3.01 ± 0.3 ^a,c,e^	4.22 ± 0.4 ^b,d,f^	2.55 ± 0.3 ^a,c,e^
*n*-6/*n*-3 PUFA ratio	2.87 ± 0.1 ^b,d,f^	4.77 ± 0.6 ^a,c,e^	3.16 ± 0.5 ^b,d,f^	5.34 ± 0.7 ^a,c,e^	3.09 ± 0.4 ^b,d,f^	5.84 ± 0.6 ^a,c,e^

Data shown as the mean ± SD and expressed as mol % of fatty acid. ^a^ Significantly different to normal weight 6th month of pregnancy; ^b^ Significantly different to obese 6th month of pregnancy; ^c^ Significantly different to normal weight 1st month of lactation; ^d^ Significantly different to obese 1st month of lactation; ^e^ Significantly different to normal weight 6th month of lactation; ^f^ Significantly different to obese 6th month of lactation. One-way ANOVA and Bonferroni test. (*p* < 0.05). The description of saturated and unsaturated FA is the same that are shown in legend of Table 3. non-OBE: women without obesity; OBE: women with obesity.

## Data Availability

All data used and/or analyzed during the study are available from the corresponding author on request.
